# Recognizing expressions of thriving among persons living in nursing homes: a qualitative study

**DOI:** 10.1186/s12912-020-00526-7

**Published:** 2021-01-05

**Authors:** Rebecca Baxter, Per-Olof Sandman, Sabine Björk, Anders Sköldunger, David Edvardsson

**Affiliations:** 1 Department of Nursing, Umeå University, Umeå, Sweden; 2 Department of Neurobiology, Care Sciences and Society, Division of Nursing, Karolinska Institutet, Huddinge, Sweden; 3 Department of Public Health and Clinical Medicine, Section of Sustainable Health, Umeå University, Umeå, Sweden; 4 Department of Neurobiology, Division of Neurogeriatrics, Care Sciences and Society, Karolinska Institutet, Stockholm, Sweden; 5 School of Nursing and Midwifery, La Trobe University, Melbourne, Australia

**Keywords:** Nursing, Thriving, Well-being, Nursing home, Staff education, Long-term care, Aged care, Person-centred care, Qualitative content analysis

## Abstract

**Background:**

Thriving has emerged as a contemporary and health-promoting concept for older people living in nursing homes; however, there has been limited research to explore how nursing home staff identify thriving in their everyday practice. The aim of this study was to explore how staff recognize expressions of thriving among persons living in nursing homes.

**Methods:**

Semi-structured interviews were conducted with 14 nurses working at a nursing home in Victoria, Australia. The interviews were audio-recorded, transcribed and analyzed using qualitative content analysis.

**Results:**

The analysis resulted in six sub-categories and three main categories. Expressions of thriving were recognized in relation to how staff understood thriving, observed thriving and sensed thriving. Staff described comparing and contrasting clinical assessment indicators with their own personal and professional understandings of thriving, as well as their overall sense of the individual person within the wider situational and environmental context.

**Conclusions:**

Our results illuminate how staff recognize everyday expressions of thriving for people living in nursing homes and emphasizes the importance of utilizing person-centred care principles in clinical assessments. These findings have practical implications with regards to how thriving is identified and assessed in long-term care, and could be used to inform and guide staff education, person-centred care strategies, and organizational policies to better support and promote thriving in nursing homes.

**Supplementary Information:**

The online version contains supplementary material available at 10.1186/s12912-020-00526-7.

## Background

The concept of thriving in nursing homes has been described as encompassing place-related well-being relative to the individual and the wider institutional, relational and environmental context [1, 2]. As the perception of what constitutes thriving is said to be largely up to the individual, experiences of ill-being (i.e. physical, functional and/or cognitive limitations) do not necessarily impede thriving as a person may simultaneously experience their life as being positive [1, 2]. Experiences of thriving for older people living in nursing homes are said to be influenced primarily by their attitude towards living in the nursing home, and the quality of their care and caregivers [1]. Other influential aspects of the social and spacial lived-environment are thought to include relationships with family, qualities in the physical environment, opportunities to get outside and around, participation in meaningful activities, and relationships with their peers [1]. Previous studies have explored the concept of thriving from the experiential perspective of people living in nursing homes; however, little is known about how staff identify everyday expressions of thriving in nursing homes, or how this compares with resident descriptions of thriving, and no studies have explored the assessment of thriving from the perspective of Australian nursing home staff.

There has been limited research regarding staff perspectives of thriving in nursing homes despite their involvement in the assessment and measurement of thriving as proxy-raters in previous studies [e.g.] [3–6]. An American study recently sought to explicate the concept of thriving in long-term care by amalgamating resident and staff perspectives into a single definition of thriving [7]. Several overlapping aspects were identified, such as engagement in activities, opportunities for involvement in decision-making and the personality of the individual. However, understandings of thriving diverged when describing aspects such as physical characteristics, as staff perceived that loss of function or independence hindered thriving, while the residents themselves articulated that they did not typically experience their health problems to be a barrier to thriving [7]. The resulting definition of thriving combined both staff and resident perspectives, leading to confusion as to which parts of the definition were reported by whom and which parts could be meaningfully used when attempting to recognize or assess expressions of thriving in practice [8].

Foundational thriving research among Norwegian nursing home residents linked experiences of thriving to relationships with caregivers [9] and peer relationships with other residents [10]. Close caregiver and peer relationships were important thriving requisites for some individuals; however, others indicated that professional and/or social relationships did not greatly impact their experience of thriving, demonstrating the existent variation of individual preferences within the unique care and social context of the nursing home environment [9, 10]. In a study involving Swedish nursing home residents, feelings of thriving were associated with visits from loved ones, communication with care providers, a pleasant physical environment, being secure and met with respect, and being satisfied with one’s life [11]. Likewise, meanings of thriving were explored among a group of Australian nursing home residents and were understood as a combination of acceptance, feeling supported, cared for and independent, opportunities to choose relationships, and feeling a sense of home [12]. These previous studies illuminated aspects that nursing home residents identified as being important to experiences, feelings and meanings of thriving; however, it is imperative to also consider the ways in which nursing home staff recognize these expressions in practice given that individual experiences and staff evaluations of other well-being and quality-related concepts have been found to be inconsistent or incongruent [13–15]. Therefore, this study aimed to explore how staff recognize expressions of thriving among persons living in nursing homes.

## Methods

### Participants and setting

The study was conducted over a four-week period in March 2018 at a rural nursing home facility providing residential aged care, palliative care, respite care and secure dementia care in Victoria, Australia. The nursing home was located within close proximity to the local hospital and town centre. The majority of staff employed at this facility were Enrolled Nurses (EN), however there was a Registered Nurse (RN) on-site at all times. In Australia, ENs undertake a minimum of 18 months of higher education training to receive a Diploma of Nursing, and RNs complete a 3-year tertiary education program to obtain a Bachelor of Nursing. To practice in the clinical setting, both ENs and RNs must hold a valid registration with the Nursing and Midwifery Board of Australia (NMBA) and adhere to the code of conduct, code of ethics, and professional standards for practice [16].

Following initial contact with the nursing home manager, the first (RN, PhD Candidate) and last (RN, PhD, Professor) authors presented the background, methods and aim of study at the monthly staff meeting. The inclusion criteria outlined that eligible persons would (a) be aged 18 years or older; (b) have been working at the nursing home for a minimum of three months; (c) be able to read, speak, and comprehend English; (d) hold a qualification as an RN or EN; and (e) be able and willing to provide informed consent. Those who were interested in participating were encouraged to contact the nursing home manager to schedule an interview time. The nursing home manager also invited eligible staff to participate who were not present at the meeting. Reasons for non-participation were not explored. The researchers had no pre-existing relationships with participants and were not involved in the care of residents at the nursing home. All participants were provided with verbal and written information outlining the aims and methods of the study. The information statement reiterated that participation was voluntary and that all contributions would remain anonymous. Consent was obtained via a signed and dated written consent form which outlined that participants could consent to being interviewed, being audio-recorded, or both. In total, 14 staff volunteered to take part in the study, including 2 RNs, and 12 ENs. The sample was predominantly female (*N* = 12), with a mean age of 46.6 years, and between 3 and 40 years of nursing experience (mean, 21.7 years).

### Data collection

Data were collected through semi-structured interviews which were guided by the aim of the study [17] (see: Supplementary file 1 - Interview Guide). The first two interviews were conducted with the first and last authors, and all remaining interviews were conducted by the first author. To develop a common understanding of the concept of interest, participants were first asked to describe thriving (e.g. Could you tell us about what thriving means to you?). In the second phase of the interview staff were asked how they recognized whether persons residing in the nursing home were or were not thriving. Follow up questions were guided by participant responses. All interviews took place in a private room, were scheduled during daytime hours (0900–1630), and were negotiated around the participants’ work schedule and patient care. The interviews lasted between 18 and 41 min and were transcribed by the first author for analysis. Transcripts were verified against the audio for validation.

### Data analysis

Data were analyzed using qualitative content analysis with an inductive approach [18, 19]. First, the interview texts were read in their entirety to establish an overall understanding of the content. Next, the text was divided into meaning units. Meaning units were long or short sections of text that conveyed a single meaning related to the study aim. The first author condensed and coded each meaning unit according to content (Table 1). All codes were compared, and similar codes were grouped and labelled in relation to content area to develop emergent sub-categories and categories. The preliminary findings were compared with the original text to confirm their application and relevance [18]. The results were discussed by all authors resulting in minor adjustments to wording before reaching a final consensus.

**Table 1:** Example of condensation and coding process

Meaning unit	Condensation	Code
‘… So that is how you can tell, by their interaction. That is how you can see if they are thriving with other people’	You can tell by their interaction if they are thriving with other people	Interacting with other people
‘They are not going to thrive if they are not really happy about being here’	They are not going to thrive if they are not happy about being here	Being happy about living in the nursing home
‘If someone sits at a dining room table half an hour or an hour after they have finished their meal, that’s wonderful. That means that they are engaging, you see?’	Sitting at the dining room table after they have finished their meal means they are engaging	Engaging with other people

## Results

The analysis resulted in six sub-categories and three main categories (Table 2). Supporting quotations have been provided in italics to demonstrate the interview content and to support the trustworthiness of the study. As the participants were from a single nursing home in a rural town it is not possible to link the supporting quotes with each individual’s demographic information (i.e. age, number of years of experience) without risking the confidentiality of those involved. Instead, the participant number (1–14) has been indicated in brackets following each quotation.

**Table 2:** Overview of the results

Sub-Category	Main Category
Describing professional understandings of thriving	Understanding thriving
Reflecting on personal understandings of thriving	
Assessing residents’ physical and mental well-being	Observing thriving
Seeing resident participation and engagement	
Perceiving resident attitudes towards living in the nursing home	Sensing thriving
Feeling that residents are content and satisfied	

### Understanding thriving

#### Describing professional understandings of thriving

Staff understood thriving as a positive concept that was underpinned by implicit and explicit expressions of growth, development or contentment across multiple dimensions of personhood. Staff frequently used metaphors to describe their understanding of expressions of thriving, for example one person explained *‘I think it means growing. You just are blossoming, full of life, getting the best out of it’* (P2), while another stated *‘you don’t flourish if you aren’t thriving... So you plant the seed and the seed is going to thrive into a little seedling and the flourish is the flower on the top’* (P1). Thriving was spoken of in terms of *being happy*, *making the best of it* and *doing well*. In contrast, staff stated that persons who were not actively thriving were merely *existing*, being *on hold*, or *at a standstill*. However, it was acknowledged that thriving was something that could change over time and that persons who were not thriving could thrive again given the right circumstances. As one staff member elaborated, ‘[thriving] *is everything, the person as a whole. Their emotional health can affect their physical health as well, and vice versa. Thriving to me is something that is positive, thriving in all aspects I suppose. If one of those things were down, then it would definitely affect all of the others. Like their emotional health – they might not want to get out of bed, and that turns into physical, and then they aren’t interacting and yeah, it sort of all goes into one’* (P3).

#### Reflecting on personal understandings of thriving

Reflecting on personal understandings of thriving in the professional context seemed to provide staff with greater insight into how persons in their care thrive in the nursing home environment. In their descriptions of resident thriving, staff commonly compared the situation of persons living in the nursing home to the situation that they themselves would want based on their personal interpretations of thriving. For example, one staff member narrated, *‘some people don’t need to get out of their rooms. When I am older I am probably just going to stay in my room and binge watch TV all day’* (P8), while another mused, *‘I would hate to come in here and have to share a house with a whole bunch of people that I don’t know’* (P10). Descriptions of expressions of thriving therefore seemed to be informed not only through staff’s professional understandings, but also filtered through their own personal expectations and reflections.

### Observing thriving

#### Assessing residents’ physical and mental well-being

Staff described recognizing expressions of thriving through observing indicators of physical and mental health. These observations included the presence or absence of acute illness or injury, weight loss or gain, mobility, cleanliness and anxious or depressive behaviors. For example, physical appearance seemed to be important when observing thriving, as one staff member recalled, *‘some of them can come in in a very frail state, emaciated, not eating. You know, they have lost a lot of weight, they have been isolated, they’ve got no clothes, or limited clothes that are not suitable. They come here and after about six months you will notice they’ve flourished, or thrived I suppose’* (P1). Similarly, mental well-being was also assessed through nurses’ observations, as one staff member reflected, *‘you do get residents who come in here and they just, they see this as the end point, they don’t enjoy anything and they don’t want to do anything anymore … if they have made up their minds and they don’t want to continue then it is hard to make them thrive’* (P3). Interestingly, staff outlined that residents could be observed to thrive physically, but not mentally, and vice versa. Negative symptoms of physical and mental health were observed to have an impact on thriving, however, persons could still be observed to thrive with support from other people and utilization of resources within the nursing home.

#### Seeing resident participation and engagement

Recognition of thriving also encompassed observing how residents interacted and engaged with activities, other people and the wider nursing home environment. As one staff member described *‘you find the ones that thrive the most are the ones that still get out and about and still get out in the community to do things … They know what everyone is doing, you know, they seem like they enjoy it. If they get themselves out there, join in on activities, go on bus trips, so get out there and embrace it I guess, instead of closing themselves off’* (P8). Seeing residents participating in activities and having positive interactions with others was described as being indicative of thriving. Conversely, if residents were seen to be withdrawn or isolated it was perceived as being indicative of lack of thriving. One staff member reflected, ‘*I would say it is the minority that don’t thrive, not the majority. I think it is the minority, I really do. You find that when people first come in, that is when they are very withdrawn and everything … and then you see them come out. Once they go out and go to a few activities and get involved and meet a few people, they do, they start to thrive’* (P7).

### Sensing thriving

#### Perceiving resident attitudes towards living in the nursing home

Perceiving resident attitudes about living in the nursing home was recognized as important to recognizing expressions of thriving. Staff appeared to compare and contrast their knowledge of the individual person against the ‘sense’ of congruence with their overall understandings and observations of thriving. In this way, staff outlined that recognition of thriving extended beyond what could be observed, to what could be intuited. When visual information did not match a staff member’s perception of the resident’s situation, staff indicated that they relied on their senses, for example, *‘some people appear to be happy but they are not, but other people don’t smile or they aren’t chatty or they are shy, so they might come across as withdrawn. So I don’t think you can just go on what they look like’* (P14). Acknowledging the existence of individual variations was described as important to sensing thriving, as thriving may manifest in different ways among different people depending on their disposition and circumstances. As one staff member outlined, *‘they are not going to thrive if they are not really happy about being here. If they are not choosing to be here you can see the resistance’* (P1). Being close to residents, having an open dialogue, and developing relationships assisted staff to differentiate positive or negative attitudes towards living in the nursing home.

#### Feeling that residents are content and satisfied

Staff explained that persons who were thriving tended to express contentment and satisfaction with their life situation and environment. This was described in terms of sensing that residents were accepting of their circumstances and were seemingly at home in the nursing home. As one staff member clarified, *‘you can pick up a lot by sitting and having a conversation with someone … you go in and have a chat with them and they engage with you and they are laughing and they are talking to you and they are showing you pictures of their grandkids … then you know that they are thriving doing what they are doing. They are happy, they are content … While others you might go in and they are sitting in their chair and they don’t have the telly on and they are just in the dark and you know that they are not thriving’* (P7). While visual indicators could inform features of contentment, satisfaction, acceptance and/or at homeness, staff indicated that oftentimes they simply *felt* or *knew* whether someone was or was not thriving in these aspects. In this way, staff spoke of honing and trusting their intuition while also developing their understanding of the individual person within the unique nursing home context.

## Discussion

The aim of this study was to explore how staff recognize expressions of thriving among persons living in nursing homes. Our findings revealed that thriving was recognized through a combination of *understanding*, *observing* and *sensing*. This was informed through staffs’ reflections on thriving, their knowledge of the individual person, and the overall congruence of these perceptions with their interpretation of the situational and environmental context. These categories articulate practical and descriptive examples that could be used to inform education and person-centred care strategies to enhance and promote thriving in nursing homes.

Nursing home staff are said to be well placed to make clinical assessments and judgements regarding resident experiences as they play a central role in the daily lives of persons residing in nursing homes [9, 11, 20]. Despite this, little is known about the ways in which staff inform assessments of concepts such as thriving. As reported by Sullivan and Willis [7], American nursing home staff described observing resident interactions, engagement, physical appearance and movement as being indicative of thriving. Sullivan and Willis [7] also outlined that nursing staff identified thriving when residents showed motivation to do things, wanted to get out of bed and interact with others, expressed optimism, and were perceived as being friendly or having a sense of humor. The findings from our study further elucidate the ways in which staff identify both clinical and conceptual expressions of thriving, and provides new insights into how staff process this information relative to their own reflections, understandings and experiences. For instance, familiarity and closeness with residents was said to enhance the potential for identification of expressions of thriving, or changes in thriving. Previous research has also emphasized the importance of strong interpersonal relationships from the residents’ perspective as contributing to higher perceived quality of care and feelings of acknowledgment [21]. This seems to highlight the benefits of incorporating person-centred care principles in everyday assessments and providing continued education to cultivate compassionate and empathetic interpersonal and communication skills [22–24].

While it is not possible for healthcare professionals to claim to know exactly how persons in their care experience reality, it is important to consider how clinical assessments and judgments are informed. As reported in relation to other concepts such as quality of life, over- or underestimations can have a significant impact on care delivery, decision-making and resource allocation, [e.g. 13, 14, 15]. When comparing the congruence of individual and staff perceptions of thriving, several areas appeared to correspond, namely, relationships with care staff, promotion of a sense of autonomy, engagement in activities, connections with other people and the community, and attributes within a person’s disposition [7, 9–12]. While it is encouraging that shared understandings exist around some of these aspects, our findings revealed that staff included dimensions within their assessments that were not necessarily present when the individuals themselves were asked to describe meanings and/or understandings of thriving. These included aspects such as physical appearance, behavior or even environmental characteristics. For example, staff described that smiling could be viewed as a positive expression of thriving, while sitting in a dark room could be viewed negatively in relation to thriving; however, staff did not examine these expressions in isolation, rather, they considered numerous influencing factors and made interpretations based on their knowledge of the concept, the person, the situation and the environment. As a result, potential expressions and understandings of thriving merit further comparison if such assessments are to meaningfully inform care strategies to optimize thriving.

Nursing home staff described that recognition of thriving extended beyond what could be seen, to encompass what could be felt. The use of intuition in clinical assessments has been discussed as both a type of knowledge and a way of thinking [25], and is thought to involve a level of fluidity in the movement from conscious analytic and sub-conscious thinking modes [26]. This reasoning process occurs within the continuum of a person’s cognitive processes, and is influenced by a variety of factors including knowledge, experience, exposure, rationality, matching and recognition of patterns [27–29]. Our results underscore the importance of developing skills such as insight and receptivity when conducting clinical assessments [30]. This process could be augmented by getting to know the person, building therapeutic relationships and striving to be attentive and responsive to the individual’s needs [23, 31]. In gaining a deeper understanding of the various assessment indicators related to thriving, nursing staff may be better equipped to recognize expressions of thriving among the persons they care for, as well as inform potential person-centred interventions to support and promote thriving.

### Methodological considerations

The methods used in this study require consideration. Nursing staff were invited to volunteer to be interviewed for the study risking self-selection bias. To our knowledge this is the first study to explore recognition of thriving in this context, it therefore seemed advantageous to recruit participants who were able and willing to speak on the subject matter. Importantly, the participants reported differing genders, ages, years of experience and nursing qualifications, allowing for exploration of a range of experiences related to the study aim. As outlined by Sandelowski [32], qualitative research samples should be large enough to demonstrate varied experiences, but small enough to allow for thorough analysis of all relevant aspects of the data. The trustworthiness of the study is strengthened by the use of an inductive approach to coding and categorization, and the provision of supporting quotations to exemplify the sub-categories and main categories [18]. Given that this study was undertaken within a single nursing home in rural Australia the transferability of these findings may be limited and therefore warrants further exploration in other settings.

## Conclusion

Our study found that staff recognized expressions of thriving through understanding, observing and sensing; illuminating the ways in which staff inform their everyday assessments of thriving for people living in nursing homes. These findings are important to consider when measuring, interpreting or comparing staff and resident understandings of thriving, and may be used to inform ongoing development of staff education, clinical assessment tools and person-centred care strategies. Further research is required to explore how staff use this information to support and promote thriving in nursing homes.

## Supplementary Information

**Additional file 1:.** Interview Guide

## Data Availability

The data generated and/or analyzed during the current study are not publicly available because the data that has been used is confidential.
