# Supplementary material for: Recognizing expressions of thriving among persons living in nursing homes: a qualitative study
Source: BMC Nurs. 2021 Jan 5;20:8. doi: 10.1186/s12912-020-00526-7 (PMC7786504; doi:10.1186/s12912-020-00526-7)
Supplement: Supplementary file 1 — Additional file 1:. Interview Guide [file 12912_2020_526_MOESM1_ESM.docx]

Supplementary file 1

**Interview Guide**

Example of semi-structured interview questions.

1. Can you tell us about what ‘thriving’ means to you?
2. Can you tell us how you recognize whether someone living in this nursing home is thriving?
3. Can you tell us about someone living in this nursing home who is thriving? How do you know they are thriving?
4. Can you tell us about someone living in this nursing home who is not thriving? How do you know they aren’t thriving?
5. Can you tell us how you identify expressions of thriving? Are some expressions more important than others? How do you evaluate these in your assessment?
6. Is there anything else you would like to add?
